# The role of intestinal gases in pediatric functional constipation: a narrative review of pathophysiology and emerging therapeutics

**DOI:** 10.3389/fnut.2025.1694831

**Published:** 2026-01-02

**Authors:** Yuewen Zhou, Enfu Tao

**Affiliations:** 1 Department of Pediatrics, The First People's Hospital of Pingjiang, Yueyang, Hunan, China; 2 Department of Neonatology and NICU, Wenling Maternal and Child Health Care Hospital, Wenling, Zhejiang, China

**Keywords:** pediatric functional constipation, intestinal gas, gut microbiota dysbiosis, hydrogen, methane, breath testing

## Abstract

Pediatric functional constipation (PFC) is a prevalent gastrointestinal disorder affecting approximately 18.2% of children worldwide, characterized by infrequent or painful bowel movements without organic cause, and significantly impairing quality of life, yet its management remains suboptimal. A central problem in its management is the high failure rate of conventional therapies; notably, treatments such as laxatives fail to achieve sustained relief in about 40% of pediatric patients, highlighting the critical need to explore novel pathophysiological mechanisms and therapeutic targets. Emerging evidence now highlights gut microbiota dysbiosis and the resulting imbalances in intestinal gases—particularly hydrogen (H₂), methane (CH₄), carbon dioxide (CO₂), and hydrogen sulfide (H₂S)—as key drivers of its pathophysiology. This review synthesizes current knowledge on how microbial gas metabolism influences gut motility in PFC: elevated CH₄, produced by methanogenic archaea such as *Methanobrevibacter smithii*, strongly correlates with delayed colonic transit and symptom severity, while H₂ enhances motility, and CO₂ and H₂S exert dose-dependent effects on peristalsis and mucosal signaling. Recent diagnostic advances, including H₂/CH₄ breath testing, electronic nose (E-nose) volatile organic compound profiling, and wireless motility capsules, enable non-invasive assessment of gas dynamics and transit, supporting precision phenotyping. Therapeutic strategies targeting gas-microbiota interactions—such as methane-lowering antibiotics (e.g., rifaximin), probiotics (e.g., *Lactobacillus plantarum*), low-fermentable oligosaccharides, disaccharides, monosaccharides, and polyols (FODMAP) diets, and neuromodulation—show promise, but pediatric-specific thresholds, safety, and long-term outcomes remain underexplored. The principal novelty of this review lies in its integrative framework, combining gastroenterology, microbiology, and engineering perspectives to advance gas-targeted precision medicine in PFC. Finally, we identify critical research gaps —such as the lack of pediatric-specific diagnostic thresholds and long-term therapeutic validation—and emphasize the urgent need for longitudinal studies and multidisciplinary trials to translate these insights into meaningful clinical outcomes.

## 1 Introduction

Pediatric functional constipation (PFC) is a highly prevalent gastrointestinal disorder characterized by infrequent, painful, or difficult bowel movements without organic cause, affecting approximately 18.2% of children when diagnosed using Rome IV criteria (1, 2). However, significant geographical variations highlight the influence of local factors like diet and genetics. For example, prevalence rates of 18.6% in Curacao and 16% in Jordan demonstrate that while PFC is widespread, its burden is not uniform worldwide (3, 4). A subset of these cases progresses to childhood chronic functional constipation (CFC), which impacts up to 30% of children; notably, one-third of affected children develop persistent symptoms, including overflow incontinence, abdominal pain, and psychosocial complications such as school absenteeism and social isolation (5). These symptoms not only reduce quality of life but also increase caregiver burden but are also associated with adverse long-term outcomes. For instance, a long-term follow-up study from the Netherlands reported a 15% higher school dropout rate among individuals with a history of childhood constipation (6). Other studies have also documented enduring psychological impacts (7, 8). Despite its high burden, optimal management strategies remain elusive. Conventional treatments (e.g., laxatives) fail to achieve sustained relief in 40% of children (9), and recurrence rates exceed 50% (8). The high failure and recurrence rates are largely attributable to the fact that these conventional approaches primarily address symptoms without targeting the underlying pathophysiological mechanisms, particularly those involving gut microbiota dysbiosis and its functional outputs, such as the production of bioactive intestinal gases (10).

Clinically, PFC can be stratified into three pathophysiological subtypes by colon transit time: normal-transit constipation (the most common, 52.4%), outlet obstruction/dyssynergic defecation (25.6%), and slow-transit constipation (11). Emerging efforts to refine subtype classification using machine learning approaches show promise, particularly for refractory cases (12), but reliable phenotyping remains challenging. Diagnostic systems such as Rome III and Rome IV emphasize core symptoms (e.g., infrequent defecation, hard stools, fecal incontinence) and yield similar prevalence estimates (17.3% vs. 18.2%, respectively) (1). However, Rome IV provides more precise phenotypic characterization, which is critical for guiding targeted therapies (1). Growing evidence implicates gut microbiota dysbiosis and microbial metabolites in the pathogenesis of PFC (13, 14). The gut microbiota is a key regulator of gastrointestinal motility, and its gaseous metabolic byproducts—hydrogen (H_2_), methane (CH_4_), carbon dioxide (CO_2_), and hydrogen sulfide (H_2_S)—are increasingly recognized as potential modulators of gut motility (15–17). For example, CH₄ has been linked to slowed intestinal transit (18, 19), whereas H₂ may enhance motility (20), underscoring a dynamic interplay between gas metabolism and constipation symptoms. Yet the mechanistic roles of specific intestinal gases in modulating motility and symptom severity remain poorly defined. This review systematically synthesizes current evidence on the production, metabolism, and pathophysiological effects of these gases, with a focus on their dynamic interactions with microbial communities and intestinal motility pathways. By elucidating gas–microbiota–host crosstalk, we aim to establish a framework for gas-targeted precision medicine—including diagnostic biomarkers (e.g., breath CH_4_) and tailored interventions (e.g., probiotics or gas-modulating agents)—to ultimately improve outcomes for children with PFC.

## 2 The role of intestinal gases in gut homeostasis

Intestinal gas physiology reflects a complex, dynamic system that shapes gut motility, visceral sensation, and overall gastrointestinal health. Understanding these dynamics is essential for mechanism-based therapies in PFC.

### 2.1 Gas composition and production

Intestinal gas production is a normal physiological process. Intestinal gases consist primarily of N₂, O₂, CO₂, H₂, and CH₄, with trace amounts of H₂S and other volatile organic compounds (VOCs). In healthy individuals, a dynamic equilibrium is maintained between production and elimination (21, 22): approximately 30–50% of gas is absorbed into the circulation, 10–20% is metabolized by intestinal microbes, and the remainder is expelled (15, 16). These gases originate from swallowed air (the main source of N_2_ and O_2_), microbial fermentation of undigested substrates (e.g., fibers, lactose), chemical reactions such as acid–bicarbonate neutralization in the duodenum, and trans-mucosal diffusion (17, 21). Colonic microbial activity is the principal source of H_2_, CH_4_, and H_2_S. H_2_ is generated by bacteria such as *Bacteroides* and *Prevotella* during carbohydrate fermentation. CH_4_ is produced by *methanogenic archaea* (e.g., *Methanobrevibacter smithii*) that utilize H_2_ and CO_2_, while CO_2_ arises from both microbial metabolism and chemical neutralization reactions (16, 17, 21).

The composition and volume of intestinal gases vary with diet, microbial ecology, and host physiology. Diet is a major determinant of gas profiles: high- fermentable oligosaccharides, disaccharides, monosaccharides, and polyols (FODMAP) intake increases H_2_ and CH_4_ production, whereas sulfur-containing amino acids elevate H_2_S (21, 23). In healthy individuals, intraluminal gas volume typically ranges from 100 to 200 mL, with daily production around 700 mL, most of which is absorbed or expelled as flatus (23). Disordered fermentation, such as those seen in irritable bowel syndrome (IBS) and small intestinal bacterial overgrowth (SIBO), can drive excessive gas production or retention, contributing to bloating, abdominal pain, and altered bowel habits (16).

**Figure 1:**
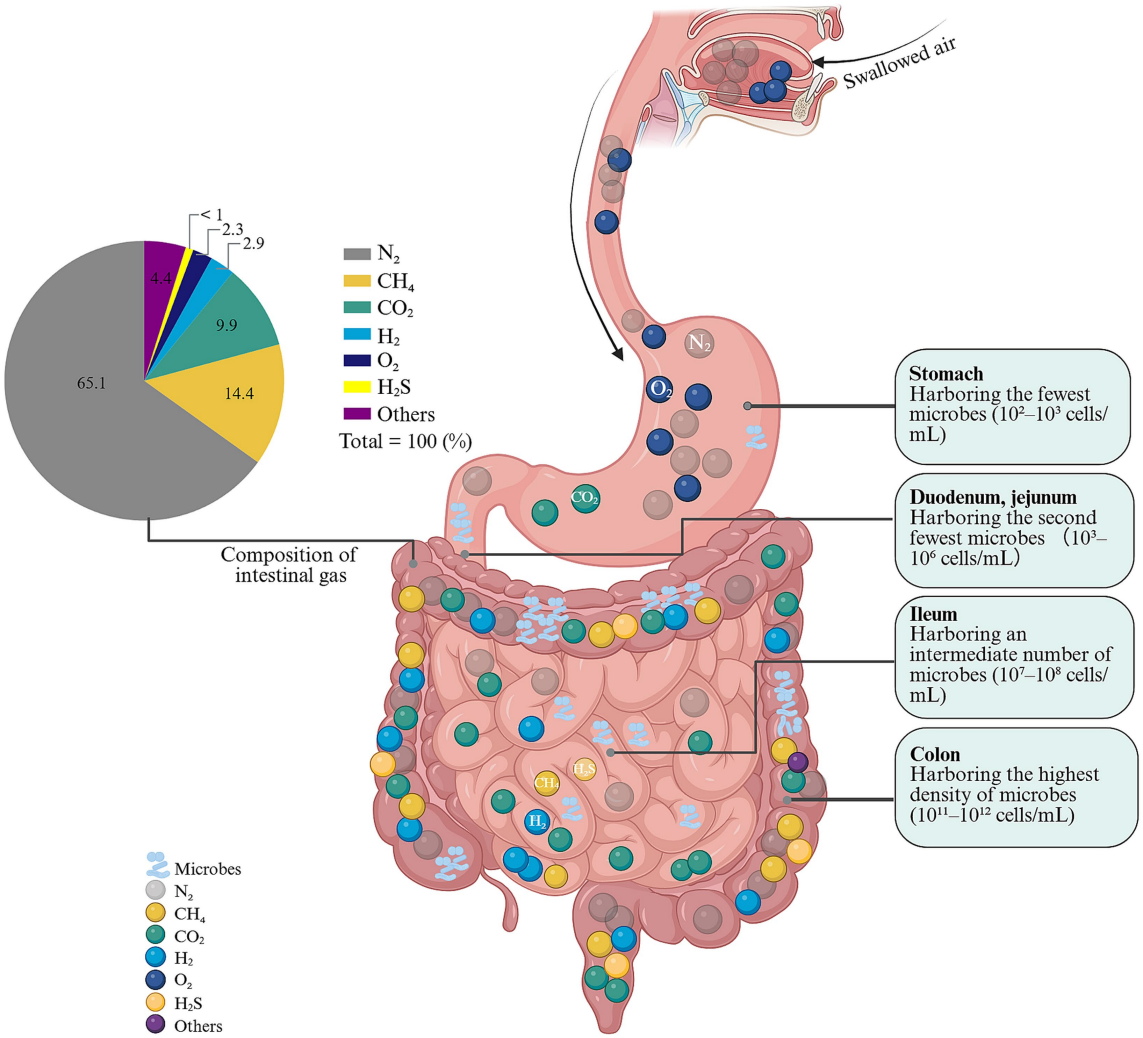
Composition and distribution of intestinal gases and microbial communities along the human digest tract. A pie chart in the upper left corner details the composition of typical intestinal gas, consisting primarily of N₂ (65.1%), followed by CO₂ (14.4%), H₂ (9.9%), CH₄ (2.9%), H₂S (<1%), and other minor components (2.3%). The central schematic illustrates the digestive tract, with each section annotated according to its microbial density and gas origins. The stomach, with the lowest microbial load, contains N₂ from swallowed air and residual O₂, while CO₂ is produced by the reaction of gastric acid with food or bicarbonate. Moving downward, the duodenum and jejunum show continued CO₂ generation from acid neutralization, with minor microbial fermentation beginning to contribute. H₂ first appears in the ileum, resulting from bacterial fermentation of carbohydrates, and microbial density increases to an intermediate level (10^7^–10^8^ cells/mL). The colon hosts the most abundant and diverse microbial community (10^11^–10^12^ cells/mL), where gases are predominantly generated through microbial metabolism: H₂ is produced by fermentative bacteria (e.g., *Bacteroides*), CH₄ by *methanogenic archaea* (with high interindividual variation), and trace H₂S by sulfate-reducing bacteria. Swallowed N₂ persists but is partially absorbed. N₂: nitrogen; CO₂: carbon dioxide; H₂: hydrogen; CH₄: methane; H₂S: hydrogen sulfide; O₂: oxygen.

Maintaining a balance between gas production and consumption is critical for gut homeostasis. Microbial cross-feeding networks regulate H_2_ availability and support efficient fermentation, while short-chain fatty acids (SCFAs) generated during fermentation fuel colonocytes and influence motility (15, 24). Gaseous metabolites also participate in gut–brain and immune signaling (15, 24). H_2_-consuming bacteria and methanogens are key sinks that modulate H_2_ levels (25), and H_2_S exerts dose-dependent effects— it can be toxic at high concentrations but supports motility and mucosal defense at physiological levels (26). A nuanced understanding of these interactions can guide dietary and therapeutic strategies that modulate microbial activity and gas handling to alleviate gas-related symptoms and improve gut function. The composition and origin of intestinal gases vary significantly along the gastrointestinal tract, as summarized in Figure 1. This spatial heterogeneity underscores the importance of considering the specific site of gas production when evaluating its pathophysiological impact in disorders like PFC.

### 2.2 Intestinal microbiota and individual gut gases

The human gastrointestinal tract hosts a highly diverse microbial community—the intestinal microbiota—that is integral to gut homeostasis, including the regulation of intestinal gas production (27–29). Through intricate metabolic interactions with dietary substrates and the host, this ecosystem generates a range of gases that are essential for normal gut function (30). Microbial density increases progressively along the gastrointestinal tract: the stomach contains the fewest organisms (approximately 10^2^–10^3^ cells/mL), followed by the duodenum and jejunum (10^3^–10^6^ cells/mL). The ileum shows a marked rise (10^7^–10^9^ cells/mL), and the colon harbors the highest densities (10^9^–10^12^ cells/mL), underscoring the colon’s central role in fermentation and gas production (16). Within this environment, gases such as H_2_, CH_4_, and CO_2_—along with trace gases like H_2_S and nitric oxide (NO)—are produced primarily via bacterial fermentation of dietary components and through host–microbe chemical interactions (16). In human colonic gas, the five most prevalent constituents are nitrogen (N_2_) (65.1 ± 20.89%), oxygen (O_2_) (2.3 ± 0.98%), CO_2_ (9.9 ± 1.6%), H_2_ (2.9 ± 0.7%), and CH_4_ (14.4 ± 3.7%). While nitrogen and oxygen largely reflect swallowed air, H_2_, CH_4_, and CO_2_ arise predominantly from microbial metabolism, with their relative proportions shaped by diet, microbial community structure, and host physiology (16, 27–29).

### 2.3 Hydrogen

Molecular H2 is the smallest gas molecule and readily permeates cellular membranes, diffusing throughout the body (31). Once considered biologically inert, it is now recognized as a bioactive, diffusible gas with anti-inflammatory, selective antioxidant, antiapoptotic, immunomodulatory, and metabolic regulatory properties, largely derived from commensal gut bacteria (31). H_2_ is a major byproduct of microbial fermentation of host-indigestible carbohydrates (e.g., dietary fibers, oligosaccharides, resistant starch) (32). More than 70% of colonic microbes—predominantly *Bacteroidetes* and *Firmicutes*—are H_2_-producing bacteria (HPB) that express hydrogenases and generate substantial quantities of H_2_, estimated at roughly 10 liters per day in healthy young adults (33, 34). Owing to its small size and lipid solubility, H_2_ diffuses rapidly across biological membranes. Within the lumen, a large fraction is consumed by hydrogenotrophic microorganisms, which convert H_2_ into other metabolites: acetate via reductive acetogens, CH_4_ via methanogenic archaea, and hydrogen sulfide (H_2_S) via sulfate-reducing bacteria (35). Thus, the balance between H2 production and hydrogenotrophic consumption is a central determinant of luminal gas ecology and downstream host effects. A portion of luminal H_2_ is absorbed through the intestinal epithelium, enters the circulation, and distributes systemically—including across the blood–brain barrier—where it selectively scavenges cytotoxic reactive oxygen species (particularly •OH and ONOO−) within mitochondria and other compartments, while sparing physiological redox signaling (31). H_2_ also modulates inflammatory and stress-response pathways, including inhibition of nuclear factor-κB (NF-κB) signaling, suppression of NLRP3 (NACHT, LRR and PYD domains-containing protein 3) inflammasome activation, and activation of the nuclear factor erythroid 2-related factor 2 (Nrf2)/heme oxygenase-1 (HO-1) pathway, thereby enhancing endogenous antioxidant defenses and reducing pro-inflammatory cytokine release (31). Excess H_2_ not metabolized or utilized is eliminated primarily via exhalation and flatus (16, 36). Given its gut origin and detectability in exhaled breath, breath H_2_ is a principal clinical biomarker for carbohydrate malabsorption and small intestinal bacterial overgrowth (37). SIBO denotes an abnormal increase in small-intestinal bacterial load and is associated with diverse gastrointestinal symptoms (38). Overall H_2_ bioavailability reflects the dynamic interplay of production, consumption, and elimination, which in turn influences therapeutic potential across diseases and serves as a proxy for microbiota stability (39, 40). Accumulating evidence links microbiota-derived H_2_ to clinical phenotypes. H2 can enhance intestinal motility, suggesting therapeutic promise for constipation (20). Elevated H2 levels are reported in individuals with the diarrheal IBS phenotype (41), whereas inadequate H_2_ production may contribute to Parkinson’s disease pathogenesis (42, 43).

### 2.4 Methane

CH_4_ is a prominent gaseous metabolite in the human gut, produced primarily by *methanogenic archaea*, with *Methanobrevibacter smithii* being the most prevalent species (44, 45). These *methanogens* are oxygen-sensitive archaea adapted to anaerobic niches such as the gastrointestinal tract (46). Because CH_4_ is produced exclusively by gut microbes and expelled in breath and flatus, exhaled methane provides a practical, noninvasive indicator of intestinal methanogenesis (47). In humans, CH_4_ production occurs mainly in the distal colon, with additional production in the distal ileum—particularly relevant in SIBO (48–50). Approximately 30–50% of healthy adults have detectable breath methane (51). Methanogens convert H_2_ and CO_2_ to methane via the reaction 4H_2_ + CO_2_ → CH_4_ + 2H_2_O (20). This process unfolds within a competitive H_2_ economy, where *methanogens* vie with sulfate-reducing bacteria (SRB) and acetogens for molecular hydrogen (52). Intestinal methanogenesis varies with host, microbial, and environmental factors, including ethnicity, gastrointestinal disorders, gut transit, and overall community composition, with H_2_ competition from nitrate-, sulfate-reducing, and acetogenic bacteria shaping flux through methanogenesis (47, 53). Additional modifiers include bile acids, body mass index (BMI), antibiotics, sanitation, genetics, and sex (47). Age is a notable determinant: infants typically lack detectable CH_4_ through the first 6 months, even though H_2_ can be detected within 24 h after birth (47, 49). In a cross-sectional cohort of 428 healthy individuals (ages 4–95 years), methane producers increased with age across 15-year strata—0% (0/3) at 1–5 years, 5.6% (3/54) under 15 years, and 57.1% (12/21) over 75 years—demonstrating a strong age-dependent rise in prevalence (47). Another study detected breath CH_4_ in 77% of adults (122/159), 40% of older children (2–6 years; 19/47), and 8% of young children (<2 years; 4/68) (53). Diet is a key driver: higher fiber and resistant starch (RS) intake promote methane production, likely via prebiotic effects that enrich methanogen-supporting networks (54, 55). In lactase-deficient children, unabsorbed lactose fermentation increases CH_4_ to 5.1 ppm at 90 min, versus 1.6 ppm with normal lactase activity, underscoring lactose malabsorption as a contributor to methanogenesis (56).

The impact of CH₄ on intestinal motility presents a compelling, though complex, picture. A body of evidence supports its inhibitory role. In animal models, CH_4_ slows peristalsis by increasing contraction amplitude and prolongs small-intestinal transit time by 59% (57). In humans, CH_4_ producers frequently display prolonged colonic transit, and case imaging with radioactive markers has linked retained colonic markers at 60 h to elevated breath methane, consistent with slow-transit constipation (58). Clinically, CH_4_ is associated with functional constipation and constipation-predominant IBS-C. A meta-analysis of 1,277 participants showed methane positivity correlated with IBS-C (OR 3.51; 95% CI, 2.00–6.16) (59). IBS-C patients also demonstrate increased *Methanobrevibacter smithii* abundance compared with healthy controls (60). Higher breath CH_4_ has been positively correlated with constipation severity (19), and methane positivity may reflect a blunted postprandial serotonin response, potentially contributing to impaired peristalsis (61). However, a critical interpretation of this association is warranted. The translation of these findings into a definitive pathophysiological mechanism for PFC is not straightforward. While evidence from animal models and clinical studies suggests CH₄ slows intestinal transit (57, 62, 63), its role in PFC requires critical appraisal. The literature reveals inconsistencies; for instance, a meta-analysis linked methane positivity to constipation (59), whereas Di Stefano et al. (64) found no significant association between colonic methane production and IBS symptoms or transit (64). This discrepancy may arise from methodological heterogeneity, particularly the pooling of IBS subtypes, which can obscure the specific CH₄-constipation relationship seen in more homogenous PFC populations.

### 2.5 Carbon dioxide

CO₂ is a critical gaseous metabolite in the human gastrointestinal (GI) tract, with roles spanning normal physiology and disease. It arises from both microbial fermentation and host metabolic processes, generated in different intestinal segments via distinct mechanisms. In the stomach and proximal small intestine, CO₂ is produced primarily through chemical reactions—most notably the neutralization of gastric acid (HCl) by dietary or pancreatic bicarbonate (HCO₃^−^) (16, 36). In contrast, in the distal small intestine and colon, microbial fermentation of undigested carbohydrates and proteins by commensal bacteria is the dominant source (16). The resulting CO₂ is passively absorbed into the circulation more rapidly than other luminal gases (H₂, CH₄, N₂, O₂) and is subsequently eliminated via the lungs (21). Although absorption is rapid, the rate of intraluminal production often exceeds local removal at the site of generation; moreover, because a substantial fraction is absorbed and exhaled, the total gas produced in the gut typically exceeds the volume expelled as flatus (65).

Diet is a major determinant: carbonated beverages and fermentable carbohydrates (e.g., lactose, fructose, dietary fibers) furnish substrates for chemical neutralization and microbial fermentation (16, 36, 66), while protein-rich diets may increase CO₂ output via bacterial deamination of amino acids (67). Impaired digestion and dysbiosis amplify fermentation: enzyme deficiencies (such as lactase insufficiency) and SIBO divert undigested substrates to distal segments, markedly increasing CO₂ yield (68, 69). Microbiota composition matters: the abundance and metabolic activity of gas-producing taxa, including *Bacteroides*, shape fermentation efficiency and CO₂ output (70). Overall, intraluminal CO₂ levels reflect a dynamic balance between production (dietary, microbial, metabolic) and elimination (absorption and excretion); disturbances on either side can lead to symptomatic gas retention—bloating, belching, and flatulence.

Traditionally regarded as inert aside from volume-related mechanostimulation (16), CO₂ also demonstrates therapeutic activity in GI disorders via synergistic mechanisms. In functional dyspepsia and constipation, CO₂ delivered as carbonated water can alleviate symptoms through: (1) chemical activation of trigeminal and vagal pathways via carbonic acid–mediated pH changes, modulating visceral sensitivity; (2) mechanical distension of the proximal stomach, enhancing gastric accommodation and reducing early satiety; and (3) mineral-mediated stimulation of gallbladder contraction. These benefits occur without accelerating GI transit, indicating primarily neuromodulatory rather than prokinetic effects (71). However, the evidence supporting this therapeutic use is derived from small-scale adult studies, and its efficacy and safety in the pediatric population remain entirely unexplored. In addition, while CO₂ exhibits anti-inflammatory actions in colitis models, suppressing pro-inflammatory cytokines (e.g., IL-6) and preserving mucosal integrity through enhanced mucin (MUC-2) secretion (72), it remains speculative whether these mechanisms operate in non-inflammatory context of functional constipation. Together, the convergence of mechanical, chemical, and neurohumoral effects positions CO₂ as a distinctive therapeutic modality for sensory and motor dysfunctions in the GI tract.

### 2.6 Hydrogen sulfide

H₂S is a microbiota- and host-derived gasotransmitter that is essential for gut homeostasis, acting at the interface of microbial ecology and mucosal defense (73–75). It is produced by sulfate-reducing bacteria (e.g., *Desulfovibrio*) via dissimilatory sulfate reduction and by host epithelial cells through the enzymes cystathionine *β*-synthase (CBS) and cystathionine *γ*-lyase (CSE). By coordinating signals between microbes and the epithelium, H₂S helps shape community structure and maintain barrier function. Mechanistically, H₂S stabilizes the microbiota biofilm and preserves the mucus layer, physically separating microbes from the epithelial surface (73, 76). This barrier function is crucial for preventing bacterial translocation and maintaining epithelial integrity. Consistent with this, H₂S donors restore biofilm organization and enhance mucus production in colitis models (73, 76).

The regulation of H₂S production is tightly coupled to microbial ecology. The human gut microbiota broadly retains the capacity to generate H₂S from cysteine, and dysbiosis often coincides with elevated H₂S output (77, 78). Increases in H₂S-producing taxa, such as *Bilophila wadsworthia*, have been associated with inflammatory bowel disease (IBD) and certain IBS subtypes. Under these conditions, excess H₂S can disrupt disulfide bonds within mucus, leading to epithelial injury (78–80). However, it is critical to note that the majority of evidence linking pathogenic levels of H₂S to mucosal damage originates from studies on IBD or severe IBS. The role of H₂S in the relatively non-inflammatory context of PFC remains far less clear and is primarily extrapolated from these related conditions, representing a significant knowledge gap. Furthermore, the evidence presents a paradoxical, dose-dependent role for H₂S. At physiological levels, H₂S exerts anti-inflammatory effects by limiting neutrophil infiltration and modulating redox signaling within the lamina propria (76, 81).

Dietary inputs strongly modulate H₂S dynamics. Protein-rich diets tend to enhance microbial H₂S production, whereas dietary fiber can mitigate this by promoting SCFAs formation and lowering luminal pH (82, 83). This modulation is clinically relevant, as H₂S metabolism is frequently dysregulated in pediatric gastrointestinal disorders (80). Nevertheless, direct interventional studies examining the impact of dietary modulation on H₂S levels and subsequent symptoms in children with PFC are lacking. Most dietary recommendations are inferred from studies on adult IBS or animal models, highlighting the need for pediatric-specific nutritional research. At the ecosystem level, bacterial H₂S supports cryptic redox reactions that shape microbial niches, underscoring its central role in maintaining community balance (84). Dysregulated H₂S production and metabolism may contribute to functional gastrointestinal disorders, positioning H₂S-targeted strategies as promising approaches to restore microbial equilibrium and mucosal health (74, 85). However, translating this promise into clinical practice for PFC requires a more nuanced understanding. Future research must prioritize defining the concentration-dependent effects of H₂S in the developing gut, establishing reliable methods for its non-invasive measurement in children, and conducting controlled trials to determine whether modulating H₂S signaling—through diet, probiotics, or novel therapeutics—can effectively and safely improve motility outcomes in pediatric patients.

## 3 Dysbiosis, gas imbalance and pediatric functional constipation

The pathogenesis of PFC reflects an interplay of genetic susceptibility, lifestyle factors (low fiber intake, sedentary behavior), and psychological comorbidities, with a prominent contribution from brain–gut axis dysregulation manifesting as altered rectal sensitivity and pelvic floor dysfunction (86). Central to this discussion, gut microbiota dysbiosis and imbalances in microbial gases—particularly elevated CH₄ with altered H₂—emerge as key drivers of symptom onset and persistence (13, 87–90). Pantazi et al. reported that infants with functional constipation show reduced acidifying taxa (*Lactobacillus*, *Bifidobacterium*) and increased coliforms (*Escherichia coli*, *Klebsiella*), correlating with higher fecal pH and intermediate dysbiosis indices; cesarean delivery and formula feeding are risk factors (13). A consistent reduction in *Lactobacillus* has been replicated in constipated children (87). Loss of SCFAs-producing *Lactobacillus*/*Bifidobacterium* and overgrowth of proteolytic species elevate fecal pH (14), creating a niche favorable to *methanogenic archaea* that prefer neutral–alkaline conditions (pH 6.8–8.5) (91). Diminished SCFAs production is mechanistically linked to impaired motility and constipation (14). Beyond acidification and SCFAs, bile acid metabolism represents another crucial pathway through which the gut microbiota influences intestinal motility and fluid balance. While the fecal bile acid profile is normal in the majority of children with functional constipation, a subset of patients exhibits a novel abnormality characterized by extensive sulfation of bile acids. Hofmann et al. (63) identified that approximately 8% of constipated children (6 out of 73) had fecal bile acids predominantly composed of the 3-sulfate of chenodeoxycholic acid (CDCA). As sulfation abolishes the secretory activity of CDCA, this alteration in bile acid metabolism may directly contribute to reduced colonic secretion and worsened constipation symptoms in this patient subgroup, highlighting another microbial metabolic pathway implicated in PFC pathogenesis. Elevated CH₄ correlates with delayed transit and constipation across pediatric cohorts. CH₄-positive children show prolonged total colonic transit (median 80.5 vs. 61.0 h; *p* = 0.04) (62); a meta-analysis found CH₄-positive individuals are >3 times likelier to be constipated (pooled OR 3.51; 95% CI 2.00–6.16) (59). CH₄ excretion is more prevalent in children with encopresis than in controls (65% vs. 15%; *p* < 0.001) (92). In myelomeningocele, CH₄ producers exhibit delayed OCTT and fewer evacuations (93). In pediatric IBS, CH₄ correlates positively with whole-intestinal transit time and inversely with bowel movement frequency (94). These consistent associations across diverse patient groups strongly implicate methane as a key player in slowed gut transit. However, a critical distinction must be made between correlation and causation. It remains plausible that the prolonged transit time inherent to constipation itself creates a favorable anaerobic environment that enriches for methanogens, rather than methanogenesis being the initial trigger. The mechanistic link is supported by experimental evidence: CH₄ directly slows motility—reducing ileal peristaltic velocity (~20%) while increasing contraction amplitude and intraluminal pressure in preclinical models—whereas H₂ accelerates proximal colonic transit (~47% decrease in time) (20). Pimentel et al. showed methane impedes small-intestinal transit and augments contractility (57). Similarly, Zhang et al. (95) demonstrated that *Lactobacillus plantarum* Lp3a significantly mitigated functional constipation in both murine and human subjects by promoting intestinal motility, potentially through the modulation of CH₄ and bile acid metabolic pathways. Electro-acupuncture restored the *Firmicutes*/*Bacteroidetes* ratio and increased butyrate, enhancing motility (96). Consistently, butyrate levels negatively correlate with methanogen abundance, implying competition for H₂ that can be steered toward SCFAs rather than CH₄ production (91). The competition for metabolic substrates, such as H₂, between SCFAs-producing bacteria and methanogens further substantiates the hypothesis that enhancing butyrate levels—through interventions such as probiotics, including *Lactobacillus* species, or electro-acupuncture—may inhibit methanogenesis, thereby improving gut motility and alleviating constipation. Probiotics such as *Lactobacillus casei rhamnosus* Lcr35 may improve motility via GPR41 signaling by increasing SCFAs (97), though whether Lcr35 directly suppresses *methanogens* and lowers CH₄ remains uncertain and warrants study. CH₄-driven dysbiosis is best characterized as intestinal methanogenic overgrowth (IMO)—overgrowth of archaea (notably *Methanobrevibacter smithii*)—rather than classic SIBO (98, 99). CH₄-dominant small intestinal bacterial overgrowth (M-SIBO) is associated with slower small-bowel and colonic transit than H₂-dominant SIBO (H-SIBO) (100). Prolonged lactulose breath test patterns mirror delays in small bowel transit time (SBTT), colonic transit, and whole gut transit time (WGTT) (101). Separately, CH₄ production in myelomeningocele aligns with longer OCTT (93).

Building upon the established role of CH₄ in decelerating intestinal motility, emerging evidence indicates that H₂ may also play a role in the pathogenesis of PFC through distinct yet complementary mechanisms. Vajro et al. found prolonged OCTT in constipated children on H₂ breath testing, suggesting small-intestinal dysmotility and a potential ecological shift toward methane generation as archaea consume H₂ (102, 103). Soares et al. observed that standard therapies reduce OCTT, yet children with persistently slow colonic transit (>62 h) often still require laxatives, possibly reflecting unresolved deficits in H₂ producers or excess methanogens (104). Ge et al. (105) demonstrated that fecal microbiota from constipated individuals impaired gut motility in murine models by decreasing the levels of microbial metabolites, including SCFAs and secondary bile acids. In contrast, fecal microbiota transplantation (FMT) effectively restored intestinal function, suggesting that modulation of the intestinal environment may represent a novel therapeutic strategy for the management of constipation. While powerful, these fecal transplant studies in animals, and the emerging evidence in humans, prove that the constipated microbiota is functionally capable of altering motility. Yet, they do not isolate H₂ as the sole critical factor. The therapeutic effect is likely mediated by a consortium of changes, including the restoration of SCFAs, bile acids, and other microbial signals, of which H₂ modulation may be just one component. Together, rebalancing microbiota and the H₂/CH₄ axis is a promising therapeutic angle, pending pediatric-specific validation. Key clinical studies investigating the role of intestinal gases, particularly H₂ and CH₄, in PFC were shown in Table 1.

**Table 1:** Summary of clinical evidence on intestinal gases in pediatric functional constipation.

Author, year	Study type	Study population	Intestinal gas source	Key findings	Conclusion
Vajro, P. et al., 1988 (102)	Case–control clinical	14 constipated children (2–11 years old), 11 age-matched controls (for lactulose breath test), 7 age-matched controls (for standard meal breath test)	Hydrogen	OCTT with standard meal: constipated children had longer transit time (336.9 ± 17.43 min) than controls (275 ± 15.19 min).	Small intestinal transit is delayed in constipated children
Fiedorek, S. C. et al., 1990 (92)	Clinical case–control	40 encopretic children (mean age 8.3 ± 3.0 years), 27 constipated children (mean age 7.1 ± 2.9 years), age/race/sex-matched controls	Methane	(1) 65% (26/40) encopretic children were methane producers vs. 15% (6/40) controls (*p* < 0.001); and (2) 63% reduction in methane producers post-treatment in asymptomatic encopretic patients (*p* < 0.05)	Methane production is prevalent in encopretic children and decreases with treatment; not linked to simple constipation.
Fontenele Soares, A. C. et al., 2005 (62)	Prospective clinical	40 children (3–13 years) with chronic constipation	Methane	(1) 73.5% (25/34) constipated children with soiling were producers vs. 16.7% (1/6) without soiling (*p* = 0.014); (2) Median total CTT: 80.5 h (producers) vs. 61.0 h (non-producers) (*p* = 0.04); and (3) Left colon CTT prolonged in producers (*p* = 0.001).	Methane is associated with slow colonic transit time and soiling in constipated children
Fontenele Soares, A. C. et al., 2005 (103)	Prospective clinical	34 children (3–13 years) with chronic constipation, 15 healthy controls	Hydrogen	(1) OCTT with lactulose: no difference between constipated (63.8–66.9 min) and controls (65.3 min) (*p* = 0.727); (2) OCTT with bean meal: 252.4 min (constipated with delayed CTT) vs. 205.3 min (controls) (*p* < 0.05); and (3) 61.8% (21/34) constipated children had increased total CTT (>62 h).	hydrogen breath test with bean meal detects delayed OCTT in constipated children with abnormal CTT.
Hofmann, A. F. et al., 2008 (63)	Cross-sectional clinical	207 children (1 month–12 years): 103 with functional constipation, 104 controls	Indirect (bile acid sulfation)	(1) 6 constipated children had 3-sulfate of CDCA as dominant fecal bile acid; (2) Sulfation abolishes CDCA’s secretory activity (exacerbates constipation); and (3) 94.2% (97/103) constipated children had normal bile acid profiles.	A small subset of constipated children has abnormal bile acid sulfation, indirectly disrupting gas/water balance.
Ojetti, V. et al., 2014 (93)	Prospective clinical	18 children (6 males/12 females; 16.4 ± 7.6 years) with myelomeningocele and constipation	Methane/hydrogen	(1) 44.4% (8/18) produced high methane (mean peak 25 ± 13 ppm); and (2) All methane producers had delayed OCTT.	Methane is associated with delayed OCTT, and constipation.
Chen, K. et al., 2024 (160)	Randomized controlled trial	131 children (0–6 years) with functional constipation (65 intervention, 66 placebo)	Methane metabolism-related genes	(1) Intervention group (Bifidobacterium XLTG11) had higher stool frequency (3.69–4.03) vs. 2.89–3.18 times/week, (*p* = 0.0067); and (2) methane metabolism genes downregulated; SCFAs genes upregulated	Bifidobacterium XLTG11 improves constipation and downregulates methane metabolism genes.
Pantazi, A. C. et al., 2025 (13)	Longitudinal clinical	134 infants (1–12 months): 82 with FGID (including functional constipation), 52 without FGID	Indirect (gut microbiota imbalance)	(1) FGID infants had intestinal dysbiosis (lower acidifying, higher proteolytic flora); (2) Cesarean section/formula feeding linked to higher FGID/dysbiosis risk; and (3) dysbiosis implies altered microbial gas metabolism.	Gut microbiota imbalance is key to FGID (including constipation) and indirectly disrupts gas metabolism.
Xiao, P. et al., 2025 (112)	Self-controlled clinical	54 children (6–12 years) with functional constipation	Indirect (microbiota/metabolites linked to gas balance)	(1) Dietary fiber increased stool frequency (2.2 → 5.0 times/week) and improved consistency; (2) microbiota: increased *Lachnospiraceae_ND3007_group*/*Prevotella*, decreased *Enterobacter*; and (3) Metabolomics: upregulated SCFAs metabolism (linked to normal gas production).	Dietary fiber alleviates constipation and modifies microbiota/metabolites to restore gas balance.

OCTT, orocecal transit time; CTT, colonic transit time; ppm, parts per million; SCFAs, short-chain fatty acid; FGID, functional gastrointestinal disorder; CDCA, chenodeoxycholic acid.

CO₂ is abundant in the gut (from fermentation and acid–base neutralization) and shapes gas homeostasis. Dysbiosis with fewer *Lactobacillus*/*Bifidobacterium* raises pH (13, 87), favoring methanogens that use CO₂ and H₂ to generate CH₄, thereby inhibiting peristalsis (57, 62). Conversely, exogenous CO₂ (e.g., carbonated water) can alleviate dyspepsia/constipation symptoms via vagal mechano- and chemosensory activation and anti-inflammatory effects (IL-6 suppression, mucosal protection) (71, 72). However, excess CO₂ from fermentable substrates or SIBO may worsen bloating and perceived dysmotility (16, 66, 68, 69). Strategies aimed at restoring microbial balance to divert reducing equivalents (e.g., H₂) toward SCFAs production and away from methanogenesis (91, 95), and consider controlled CO₂ delivery as a neuromodulatory adjunct (71) present promising therapeutic avenues. However, it must be critically acknowledged that the current understanding of CO₂’s net effect in PFC is rudimentary. The direct evidence linking CO₂ dynamics to pediatric constipation is scarce, and the translation of potential therapeutic uses from adult studies remains entirely speculative. Therefore, dedicated pediatric investigations are crucial to validate these mechanistic links and explore the therapeutic potential of modulating CO₂ in the management of childhood constipation.

**Figure 2:**
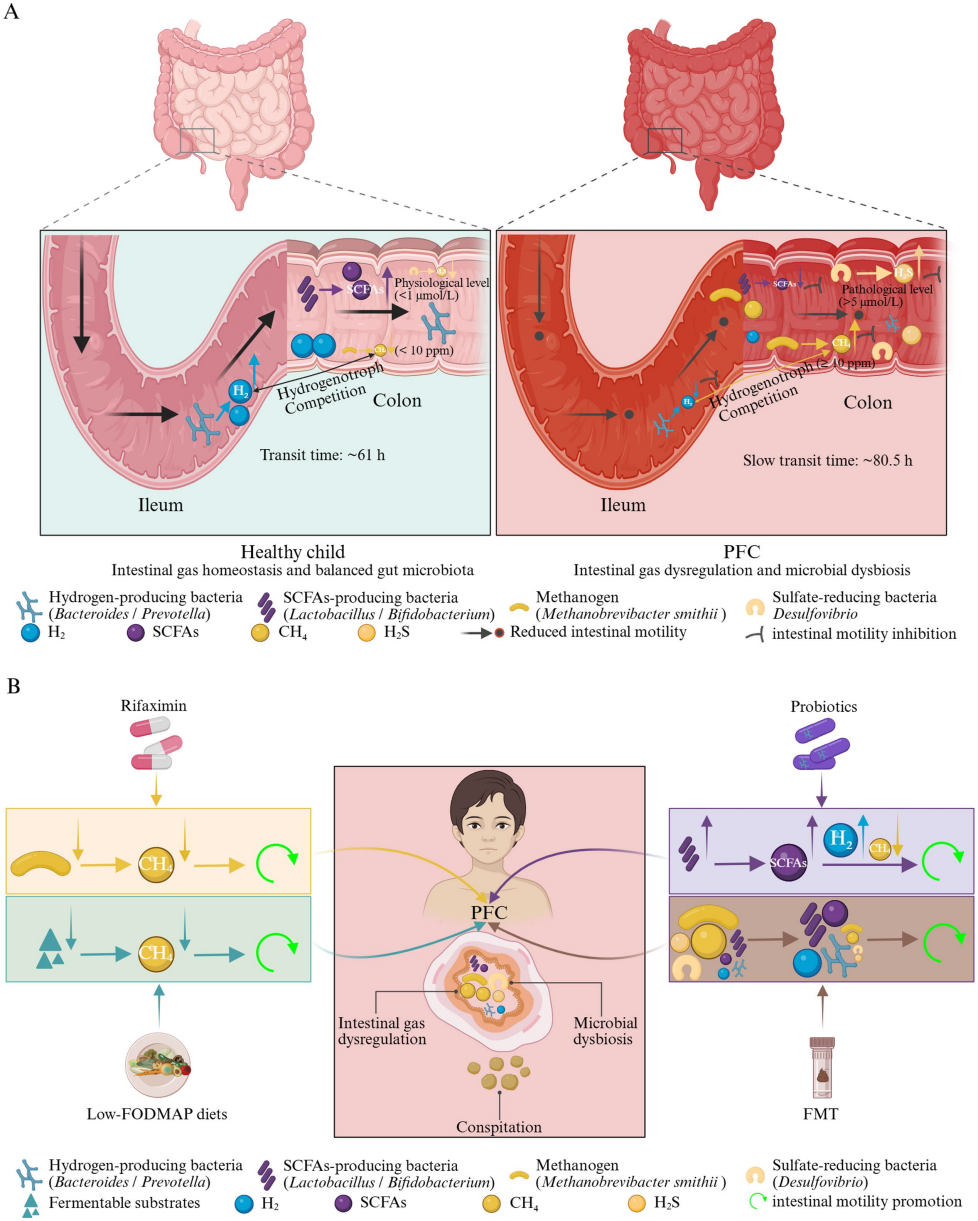
Pathophysiological mechanisms and potential therapeutic interventions in pediatric functional constipation. **(A)** Comparison of intestinal environments between a healthy child and a child with PFC. In the healthy state, balanced microbiota dominated by *Bacteroides*, *Prevotella*, *Lactobacillus*, and *Bifidobacterium* maintain physiological gas levels, including ample H₂, low CH₄ (< 10 ppm), and low H₂S (< 1 μmol/L), supporting normal colonic transit time (~61 h). In PFC, reduced abundance of H₂-producing bacteria leads to decreased H₂ levels, which alongside dysbiosis promotes overgrowth of *methanogens* (*Methanobrevibacter smithii*) that consume H₂ to produce excessive CH₄ (≥ 10 ppm). Concurrent increase in sulfate-reducing bacteria (*Desulfovibrio*) elevates H₂S (>1 μmol/L). These changes collectively inhibit intestinal motility and prolong transit time (~80.5 h). **(B)** Potential therapeutic interventions in PFC target the underlying dysbiosis and gas imbalance. Rifaximin suppresses *methanogenic archaea* to reduce CH₄ production and may indirectly modulate H₂ availability. Probiotics enrich beneficial SCFAs-producing and H₂-producing bacteria, which helps restore microbial balance and increase H₂ levels. FMT reintroduces a healthy microbial community to reestablish ecological and functional homeostasis. Additionally, a low-FODMAP diets limit fermentable substrates, thereby reducing overall gas production—including CH₄. PFC: pediatric functional constipation; H₂: hydrogen; CH₄: methane; H₂S: hydrogen sulfide; SCFAs: short-chain fatty acids; FODMAP, fermentable oligosaccharides, disaccharides, monosaccharides, and polyols; FMT: fecal microbiota transplantation.

H₂S is an endogenous and microbial gasotransmitter that exerts complex, dose- and context-dependent effects on colonic motility. It directly modulates smooth muscle by inhibiting L-type Ca^2+^ channels (CaV1.2) and BKCa channels, decreasing Ca^2+^ influx and hyperpolarizing membranes to reduce contractions (106). It shows a biphasic profile—transient excitation via transient receptor potential vanilloid-1 (TRPV1) and substance P release, followed by sustained inhibition through ATP-sensitive potassium (KATP) activation (107). An overgrowth of SRB can delay intestinal transit in a manner that is reversible with bismuth treatment, indicating that microbial dysbiosis may exacerbate symptoms associated with functional conspitatoin (108). H₂S cross-talk with Toll-like receptors (TLR2/TLR4) regulates cystathionine-*γ*-lyase (CSE) expression, establishing a neuro–immune–microbiota axis that impacts motility (109). Synergy with NO via phosphodiesterase 5 (PDE5) inhibition enhances cyclic guanosine monophosphate (cGMP)/protein kinase-G (PKG) and smooth muscle relaxation (110). These findings underscore the significance of H₂S as a key regulator of gut motility, suggesting that its dysregulation—whether due to endogenous overproduction, microbial influences, or altered receptor sensitivity—may contribute to the pathophysiology of PFC. This insight points to potential therapeutic interventions, including the modulation of SRB, inhibition of CSE, or combined modulation of the NO signaling pathway (111). However, translating these mechanistic insights into PFC therapies remains challenging. The biphasic, dose-dependent actions of H₂S complicate therapeutic targeting, and direct clinical evidence in pediatric constipation is still lacking, relying largely on inferences from animal studies or other GI disorders. Future work should prioritize validating H₂S as a clinical biomarker or target in pediatric cohorts. Gut dysbiosis and microbial gas imbalance are central to PFC. Loss of SCFAs producers and expansion of proteolytic species elevate luminal pH, enabling methanogens that convert H₂/CO₂ to CH₄, a potent inhibitor of transit. In contrast, H₂ can promote motility, and H₂S finely tunes ion-channel activity and interacts with NO pathways. Microbiota-directed and gas-targeted therapies—probiotics, FMT, dietary strategies to boost SCFAs and curb methanogenesis, neuromodulatory CO₂ delivery, and selective H₂S-pathway modulation—are promising, but pediatric thresholds, biomarkers, and long-term safety require further study. Supporting the role of dietary interventions, a recent randomized controlled trial by Xiao et al. demonstrated that supplementation with a wheat bran-derived dietary fiber (Testa Triticum Tricum Purif) significantly improved constipation symptoms in Chinese children, accompanied by increases in beneficial bacteria such as *Lactococcus* and *Prevotella*, and modulation of metabolic pathways including steroid hormone biosynthesis and alpha-linolenic acid metabolism (112). Collectively, this evidence positions the “dysbiosis-gas-dysmotility” axis as a compelling model for PFC. However, the heterogeneity of the disorder implies that successful translation will depend on identifying which patients are most likely to respond to specific, gas-targeted interventions, moving toward a more personalized therapeutic approach. The pathophysiological mechanisms linking gut dysbiosis, gas imbalance, and slowed motility in PFC, along with potential therapeutic interventions, are illustrated in Figures 2A,B. This conceptual framework visually integrates the key players—microbial shifts, gaseous metabolites, and their functional effects on motility—providing a foundation for understanding targeted therapeutic approaches.

## 4 Methods for measuring gastrointestinal gas

Accurate quantification of intestinal gases—specifically H₂, CH₄, CO₂, and H₂S—is essential for elucidating their role in physiological and pathological conditions. A variety of diagnostic methodologies, ranging from non-invasive breath tests to invasive direct sampling techniques, have been developed to evaluate gas profiles. Method selection should be tailored to patient age, clinical question, and required analytical resolution, balancing feasibility, comfort, safety, and the need for temporal or spatial detail. This section reviews established and emerging technologies, highlighting their strengths, limitations, and quality-control considerations, with a particular emphasis on pediatric applicability and adaptation.

### 4.1 Hydrogen breath test

H₂ breath testing (HBT) is a non-invasive tool to quantify microbial fermentation, carbohydrate malabsorption, and gut motility by measuring exhaled H₂ (and CH₄). Humans do not produce H₂ endogenously; all exhaled H₂ derives from anaerobic bacterial fermentation of unabsorbed carbohydrates (e.g., lactose, fructose, lactulose) (113–116). When these substrates reach the colon, commensals metabolize them to H₂, which is absorbed into blood and exhaled, providing an indirect readout of fermentation activity (103, 114). In PFC, HBT informs two domains: OCTT assessment and detection of abnormal fermentation/dysbiosis.

### 4.2 Oro-cecal transit time assessment

OCTT, defined as the duration required for a substrate to traverse from the oral cavity to the cecum, is often prolonged in PFC, which can exacerbate stool retention and lead to symptoms such as abdominal distension (103, 117). Lactulose HBT is a standard method to estimate OCTT in children. After oral lactulose, a sustained H₂ rise ≥20 ppm above baseline marks cecal arrival and OCTT completion (103, 118). Children with slow-transit constipation (STC) show significantly prolonged OCTT (median 252 vs. 205 min in controls and constipated children with normal transit), consistent with excess fermentation, gas accumulation, and distension (103, 117).

### 4.3 Detection of abnormal fermentation and dysbiosis

HBT detects carbohydrate malabsorption and SIBO. A ≥ 20 ppm H₂ rise after lactose suggests lactose malabsorption, more common in constipated children and linked to bloating (114, 119, 120). Furthermore, CH₄, produced by methanogenic archaea (e.g., *Methanobrevibacter smithii*) that utilize H₂, plays a distinct role in pediatric constipation. Constipated children frequently exhibit elevated breath CH₄ (≥10 ppm), which correlates with prolonged colonic transit and greater symptom severity (120, 121). Methane directly inhibits intestinal motility, fostering a self-perpetuating cycle of delayed transit and gas retention, thereby positioning CH₄ as a viable therapeutic target (120).

Pediatric HBT requires age-specific protocols to ensure accuracy and feasibility. Recommended dosing includes lactulose 10 g or lactose 0.5–2.0 g/kg (max 50 g), with breath samples collected every 30 min over 3–4 h to capture fermentation dynamics (114, 122, 123). In younger children, child-friendly sampling—such as face masks or nasal probes—is often necessary to obtain reliable end-expiratory samples (103, 114). Interpretation should recognize that ~10–15% are non-H₂ producers, redirecting fermentation toward CH₄ or H₂S; therefore, combined H₂/CH₄ testing improves diagnostic accuracy, as H₂S is not routinely measured (102, 103, 119, 122).

In PFC, HBT data can directly inform targeted interventions. For example, a prolonged OCTT on lactulose HBT supports the use of prokinetic therapy to address underlying motility delay (103). Elevated breath methane (≥10 ppm) can identify candidates for rifaximin, which reduces methanogen abundance, improves colonic transit, and relieves constipation symptoms (121). Documented carbohydrate malabsorption (e.g., lactose or fructose) guides dietary restriction of specific fermentable substrates, reducing gas production and discomfort and improving stool frequency and consistency. Collectively, HBT serves as a versatile tool to personalize management strategies for children with functional constipation.

Despite its utility in evaluating postprandial fermentation capacity, the HBT presents several limitations. First, methodological inconsistencies—including variation in substrate dose (e.g., lactulose 10 g vs. 16 g; glucose 50 g vs. 75 g), sampling intervals, and diagnostic thresholds—yield variable results. Higher lactulose doses can produce false positives for SIBO, whereas lower glucose doses may underdetect distal SIBO (103, 116, 124). Second, confounding factors such as recent antibiotic use (requiring a 4-week washout), high-fiber diets, smoking, and physical exercise can alter gut microbiota or gas kinetics and distort H₂ measurements (113–115). Third, about 10–15% of children are non-H₂ producers, generating CH₄ or H₂S instead; while combined H₂/CH₄ monitoring improves detection, H₂S is not routinely measured, risking false negatives (103, 117, 120). Fourth, the HBT has a limited capacity to assess segmental colonic function: it cannot distinguish slow-transit constipation from outlet obstruction, often necessitating adjunctive tests such as colonic transit studies (104, 120). Moreover, there is poor symptom–gas correlation—for example, bloating severity often fails to track measured gas levels—and pediatric feasibility issues, as young children may struggle with pretest fasting and repeated sampling, undermining data reliability (102, 115, 125). Accordingly, HBT results should be interpreted alongside clinical evaluation and complementary diagnostics when assessing PFC (103, 113, 126).

### 4.4 Methane breath testing

CH_4_ breath testing is a non-invasive diagnostic method that quantifies CH₄ in exhaled breath to assess intestinal microbial activity—particularly methanogenic archaea—and their associations with gastrointestinal disorders (44, 127). The core principle is that breath methane is not produced by human tissues; it is exclusively synthesized by anaerobic *methanogenic archaea* (e.g., *Methanobrevibacter smithii*) through fermentation of undigested carbohydrates in the gut, using H₂ and CO₂ as substrates (44, 127). CH₄ formed intraluminally is absorbed into the bloodstream, carried to the lungs, and exhaled, making measured CH₄ a reliable indicator of intestinal methanogenic activity (127, 128).

In PFC, CH_4_ breath testing serves two principal purposes: identifying methane-associated motility disturbances and assessing gut dysbiosis. CH_4_ has been implicated in the slowing of intestinal transit, a defining feature of PFC. Children with constipation frequently exhibit elevated breath methane (≥10 ppm), which correlates with prolonged colonic transit times (CTT) and more severe constipation symptoms (18, 19). Mechanistically, CH_4_ directly inhibits intestinal smooth muscle contractility, delaying transit and promoting stool retention; this vicious cycle further exacerbates constipation (19, 44). For example, a meta-analysis reported that patients with IMO had a higher prevalence of constipation (47% vs. 38%) and greater severity than those without IMO (19).

Beyond motility, CH_4_ breath testing is a valuable tool for detecting gut microbiota imbalances. *Methanogenic archaea*—the primary generators of CH_4_—compete with other H_2_-consuming microbes (e.g., SRB) for H2, thereby altering colonic fermentation patterns (127, 129). In children with PFC, overgrowth of these *archaea* can lead to excessive CH_4_ production, which slows intestinal transit and exacerbates symptoms such as abdominal bloating and discomfort (18, 130).

Conducting methane breath testing in children requires age-specific considerations to ensure accuracy. Substrate selection and dosing are weight-adjusted, with commonly used substrates including lactulose (10 g) or glucose (50 g), and breath samples collected every 15–30 min over 3–4 h (44, 115). For younger children, specialized collection tools (e.g., face masks) may be needed to ensure reliable sampling (127). Interpretation should account for age-related variability in methane production: children under 3 years rarely produce detectable methane, whereas levels increase with age and approximate adult patterns by adolescence (44, 127).

CH_4_ breath testing serves as a valuable tool for informing targeted interventions in patients with persistent PFC. Elevated methane levels (≥10 ppm) predict a favorable response to antibiotic therapy (e.g., rifaximin), which can reduce the activity of methanogenic archaea and improve gastrointestinal transit (18, 130). In parallel, dietary modification—particularly a low-FODMAP diet—reduces fermentable substrate availability, thereby lowering methane production and alleviating symptoms such as bloating and discomfort (128, 130).

Despite its clinical utility, CH_4_ breath testing has several important limitations. Methodological variability—including differences in substrate type/dose and diagnostic cutoffs for IMO (e.g., ≥10 ppm vs. ≥ 5 ppm)—can yield inconsistent results (18, 19). Confounding factors such as recent antibiotic use, dietary changes, and concurrent gastrointestinal infections can substantially alter methane output, underscoring the need for meticulous patient preparation (127, 128). Moreover, there is a limited correlation with symptom severity: some children produce little or no methane, and without concurrent H_2_ measurement, cases may be missed, increasing the risk of false negatives (44). The test also cannot localize methanogenic activity to the small intestine versus the colon, often necessitating complementary imaging or motility studies. Practical challenges in young children—including difficulties with prolonged fasting and repeated sampling—may compromise data quality, and age-related variation in methane production must be factored into interpretation. Consequently, CH_4_ breath testing should be used alongside clinical assessment and other diagnostic modalities to optimize the management of PFC.

### 4.5 Electronic nose (artificial olfaction)

An electronic nose (E-nose) is a sophisticated analytical platform that mimics the human olfactory system to detect and classify volatile organic compounds (VOCs) within complex mixtures using a multi-sensor array and pattern-recognition algorithms (131, 132). Core components include a sampling module, a sensor array (e.g., metal oxide semiconductors, conductive polymers), and a data-processing unit that translates sensor responses into distinctive “odor fingerprints” (133, 134). For clinical use, E-nose technology offers non-invasive VOCs analysis from readily accessible samples—exhaled breath, fecal headspace, and urine—making it particularly suitable for pediatric populations that require comfortable procedures (135, 136). Compared with traditional single-gas assays, it delivers comprehensive metabolic profiling by capturing the full VOCs spectrum, providing an integrated view of gut microbiota activity and host–microbiome interactions (132, 137). It also combines rapid turnaround (results within minutes) with portability for point-of-care testing across diverse clinical settings (131, 134). Its high sensitivity and accuracy derive from advanced sensor architectures (e.g., batch-uniform metal oxide nanocolumns) paired with deep learning–based algorithms, enabling precise identification of low-concentration VOCs and multi-gas recognition accuracies exceeding 98% (133). Collectively, these strengths establish E-nose technology as a rapid, accurate, and patient-friendly diagnostic tool.

In gastroenterology, E-nose technology has emerged as a transformative diagnostic tool, leveraging VOCs as sensitive biomarkers of microbial dysbiosis and metabolic disturbances (132, 138). Its clinical applications span a broad spectrum of gastrointestinal disorders, with growing evidence of significant diagnostic utility. In inflammatory bowel disease (IBD), E-nose analysis of fecal VOCs can distinguish active from quiescent disease in pediatric patients, achieving diagnostic accuracy exceeding 75% (135, 139). Innovative systems have also detected colorectal cancer by differentiating malignant from benign conditions using characteristic VOCs signatures in both fecal samples and exhaled breath (140). The technology’s reach extends to precancerous states, as shown by its ability to identify Barrett’s esophagus through distinct exhaled-breath VOCs patterns (141). In functional gastrointestinal disorders such as IBS, E-nose platforms subclassify patients based on unique VOCs profiles that correlate with microbial metabolic patterns (e.g., CH_4_ or H_2_ dominance), intestinal transit times, and symptom severity (132, 134). Collectively, these applications underscore E-nose technology’s capacity to bridge gut microbial metabolic activity with clinical manifestations, establishing it as a valuable complement to traditional diagnostics (137). By enabling rapid, non-invasive metabolic profiling, E-nose systems open new avenues for personalized diagnosis and disease monitoring in clinical gastroenterology.

In PFC, clinically meaningful subtypes include STC, normal-transit constipation, and outlet obstruction (142, 143). Mounting evidence implicates gut microbial dysbiosis in PFC pathogenesis, with constipated children showing reduced SCFAs–producing bacteria (e.g., *Lactobacillus*) and an increase in proteolytic species, changes that are linked to altered fecal pH and impaired colonic motility (14, 91). Shifts in H_2_- and CH_4_-producing taxa may further modulate motility and generate distinct VOCs signatures that could serve as diagnostic biomarkers for PFC. E-nose technology has already proven effective in distinguishing gastrointestinal disorders through VOC profiling. For example, de Meij et al. (135) demonstrated that the analysis of fecal VOCs could effectively differentiate children diagnosed with ulcerative colitis or Crohn’s disease from healthy controls, even during periods of remission, exhibiting high sensitivity and specificity. In IBS, Baranska et al. (144) identified an exhaled-breath 16-VOCs signature that distinguished IBS from health (sensitivity 89.4%, specificity 73.3%) and correlated with symptom severity. VOCs profiling has also predicted responses to low-FODMAP diets and probiotics in IBS (145). Diet–VOCs relationships are further supported by Kasti et al. (146), who reported that both a Mediterranean low-FODMAP (MED-LFD) and NICE diet reduced stool VOCs (e.g., SCFAs, branched-chain fatty acids) in non-constipation IBS, with polynomial associations between VOCs changes and symptom severity. Together, these studies underscore the feasibility of VOCs-based, non-invasive metabolic phenotyping in gastroenterology. Building on this foundation, we outline several ways in which E-nose VOCs analysis could advance the management of PFC. The technology may enable non-invasive differentiation of constipation subtypes by identifying distinct VOCs patterns. It could support continuous monitoring of microbial dynamics by tracking VOCs signatures linked to SCFAs-producing taxa, proteolytic species, and methanogenic organisms. It may also provide predictive insights into treatment response—for fiber supplementation, probiotics, or laxatives—thereby supporting personalized therapy. Additionally, serial VOCs measurements could function as objective biomarkers for disease trajectory and treatment monitoring. Taken together, this integrated, precision-medicine approach could help address current diagnostic challenges in pediatric gastroenterology. Nevertheless, current applications remain largely theoretical, and rigorous validation—including standardized sampling and analytics, pediatric reference ranges, and multicenter studies—is required before routine clinical adoption.

### 4.6 Wireless motility capsule

The wireless motility capsule (WMC) is a non-invasive, swallowable device that enables comprehensive whole-gut and segmental motility assessment by concurrently measuring intraluminal pH, pressure, temperature, and transit times (147). In PFC, it delivers objective subtype classification by accurately quantifying four key parameters: gastric emptying time (GET), small intestinal transit time (SITT), CTT, and whole-gut transit time (WGTT) (147, 148). Clinically, the WMC is particularly informative in STC, where it consistently demonstrates prolonged CTT and diminished colonic contractility on awakening, findings that support a neuropathic etiology (142).

Beyond transit profiling, the WMC helps clarify interactions between dysmotility and intestinal gas metabolism in PFC. It has identified significant associations between methane-producing archaea (e.g., *Methanobrevibacter smithii*) and prolonged CTT, consistent with CH_4_’s inhibitory effects on intestinal peristalsis (149, 150). Conversely, H_2_-dominant fermentation patterns—often linked to accelerated small-bowel transit—may aggravate bloating in mixed PFC phenotypes (151). Collectively, these insights underscore the WMC’s utility in delineating overlapping dysmotility patterns in children with complex functional gastrointestinal symptoms (152).

Compared with traditional radiopaque marker studies, the WMC offers enhanced diagnostic capabilities by enabling real-time assessment of dynamic motility parameters—including pressure amplitudes and pH fluctuations—while also demonstrating improved patient tolerance (153). Its ability to differentiate STC from outlet obstruction—for example, delayed CTT with normal rectal pressure in STC—makes it especially valuable in pediatrics, where symptom overlap complicates diagnosis (148, 154, 155). Nevertheless, clinical use requires careful consideration of age-related variability in younger children and awareness of rare technical issues such as capsule retention (156).

Future research should prioritize validating WMC-derived gas–motility correlations in pediatric populations, with particular emphasis on the role of methane in the pathogenesis of STC (157, 158). Incorporating complementary diagnostic modalities—such as breath testing and fecal microbiota analysis—may enhance the accuracy of phenotyping in PFC and facilitate the development of targeted therapeutic interventions, including agents aimed at reducing methane levels in STC (151, 159). As a comprehensive assessment tool, WMC bridges motility evaluation and metabolic insights, thereby providing a robust framework for the advancement of personalized management strategies for PFC.

## 5 Emerging gas-targeted therapeutic strategies

The evolving understanding of the “dysbiosis-gas-dysmotility” axis in PFC has catalyzed the development of novel, targeted therapeutic interventions. These strategies aim to rebalance the gut microbial ecosystem and modulate the production or effects of specific intestinal gases, moving beyond symptomatic relief to address underlying pathophysiology. Table 2 provides a comprehensive overview of the most promising gas-targeted approaches, their mechanisms, and clinical evidence.

**Table 2:** Emerging gas-targeted therapeutic strategies for pediatric functional constipation.

Strategy	Target gas/mechanism	Example interventions	Study population	Study design	Main research findings	Author, year
Methane-Lowering Antibiotics	Directly inhibits methanogenic archaea (e.g., *Methanobrevibacter smithii*); reduces CH₄ production to reverse its slowing effect on colonic transit.	Rifaximin (400 mg, three times daily for 2 weeks)	Children with CH₄-positive PFC (*n* = 13 methane producers)	Randomized double-blind placebo-controlled trial	Normalized transit: 66.7% (4/6) in rifaximin group achieved normalized colonic transit time vs. 0% in placebo. Symptom improvement: weekly stool frequency increased from 3 to 7 times/week; stool consistency (BSS) improved. Mechanistic proof: breath CH₄ levels reduced by 60.6% vs. 5.7% in placebo.	Ghoshal et al., 2018 (121).
Probiotics (Lactobacillus)	Modulates microbial metabolic pathways, primarily downregulating methanogenesis and modulating bile acid metabolism to enhance motility.	*Lactobacillus plantarum* Lp3a (2 × 10^10^ CFU/day for 7 days)	Adults with functional constipation (*n* = 120)	Randomized double-blind placebo-controlled trial	Improved outcomes: significant increases in stool frequency and improvements in stool consistency and defecation difficulty. Genomic evidence: whole-genome sequencing identified downregulation of methane metabolism pathways.	Zhang et al., 2022 (95)
Probiotics (Bifidobacterium)	Downregulates CH₄ metabolism genes and upregulates SCFAs metabolism; enriches beneficial taxa to improve gut motility.	*Bifidobacterium animalis* subsp. *lactis* XLTG11 (1 × 10^10^ CFU/day for 4 weeks)	Children with PFC (0–6 years; *n* = 131)	Multi-center randomized double-blind placebo-controlled trial	Pediatric efficacy: significantly higher weekly stool frequency and better stool consistency vs. placebo. Dual Action: downregulated CH₄ metabolism genes and enriched beneficial taxa. Safe Profile: No adverse events reported.	Chen et al., 2024 (160)
Wheat Bran-Derived Fiber	Provides fermentable fiber that enriches SCFAs-producing taxa, modulating steroid hormone and alpha-linolenic acid metabolism to improve motility.	Wheat bran fiber (3.5 g twice daily, 4 weeks)	Children with PFC (6–12 years; *n* = 54)	Self-controlled trial	Improved motility: stool frequency increased from 2.2 to 5.0 times/week; abdominal pain reduced. Microbial Shift: enriched SCFAs-producing bacteria.	Xiao et al., 2025 (112)
Carbonated Water	CO₂ forms carbonic acid, stimulating trigeminal and vagal pathways to enhance gastro-colonic reflexes and gallbladder motility.	Carbonated mineral water (1.5 L/day for 15 days)	Adults with functional dyspepsia and constipation (*n* = 21)	Randomized double-blind placebo-controlled trial	Symptom improvement: significantly reduced dyspepsia and constipation scores. Motility enhancement: improved gallbladder emptying and reduced early satiety.	Cuomo et al., 2002 (71)
Fecal Microbiota Transplantation (FMT)	Resets the gut ecosystem to normalize gas metabolism (reduce CH₄/H₂S, increase SCFAs) and restore functional microbial networks.	FMT from healthy donors (single dose + boosters)	Children with refractory slow-transit constipation (*n* = 6)	Pilot prospective study	Multi-system benefit: markedly increased stool frequency, reduced colonic transit time, and improved quality of life. Metabolic restoration: confirmed increase in fecal butyrate and decrease in CH₄ production.	Tian et al., 2016 (105)

AUC, area under the curve; BSS, Bristol Stool Scale; CH₄, methane; CO₂, carbon dioxide; CTT, colonic transit time; FMT, fecal microbiota transplantation; FODMAP, fermentable oligosaccharides, disaccharides, monosaccharides, and polyols; GIQLI, Gastrointestinal Quality of Life Index; H₂, hydrogen; H₂S, hydrogen sulfide; IBS-C, irritable bowel syndrome with constipation; PFC, pediatric functional constipation; RCT, randomized controlled trial; SCFAs, short-chain fatty acid.

CH₄-lowering interventions represent a directly targeted approach. The antibiotic rifaximin has shown efficacy in reducing CH₄ production by inhibiting methanogenic archaea, which translates to accelerated colonic transit and improved stool frequency in children with CH₄-positive PFC (121). While powerful, the long-term use of antibiotics in children raises concerns regarding microbial resistance and the sustainability of its effect, necessitating strategies to prevent recurrence. Probiotics offer a more sustainable and safer alternative for modulating the gut environment. Specific strains, such as *Lactobacillus plantarum* Lp3a and *Bifidobacterium animalis* subsp. lactis XLTG11, have demonstrated the ability to downregulate genes associated with CH₄ metabolism while concurrently enriching beneficial SCFAs-producing taxa (95, 160). This dual action not only inhibits a key constipating driver (CH₄) but also promotes pro-motility metabolites (SCFAs), making it a particularly attractive strategy for pediatric populations. Dietary modifications remain a cornerstone of non-pharmacological management. The low-FODMAP diet reduces the fermentable substrates available to gas-producing microbes, effectively lowering breath CH₄ levels and alleviating associated bloating (146). Complementarily, the supplementation with specific dietary fibers, such as wheat bran, can actively reshape the microbiota towards a SCFAs-producing phenotype, improving stool frequency and consistency (112). This suggests a nuanced dietary approach: restricting certain fermentables to reduce negative gas effects while supplementing with beneficial fibers to promote positive metabolic outputs. More invasive yet highly impactful, FMT seeks to reset the entire gut ecosystem. Early studies in refractory pediatric constipation indicate that FMT can normalize colonic transit, increase stool frequency, and crucially, shift gas metabolism by reducing CH₄ production and increasing beneficial SCFAs like butyrate (105). While promising, FMT’s invasive nature and the need for long-term safety data in children position it as a option for severe, treatment-resistant cases. Interestingly, even exogenous delivery of gases can be therapeutic. The administration of carbonated water leverages CO₂ to stimulate vagal and trigeminal pathways, enhancing gastro-colonic reflexes and improving symptoms of functional dyspepsia and constipation, although evidence remains primarily in adults (71).

In conclusion, the landscape of PFC therapy is expanding to include precision, gas-targeted strategies. The evidence summarized in Table 2 underscores a paradigm shift from one-size-fits-all laxative use to a more mechanistic, personalized approach. Future work must focus on validating these interventions in large-scale pediatric RCTs, defining patient subgroups most likely to benefit (e.g., CH₄-producers vs. hydrogen-sulfide producers), and establishing long-term efficacy and safety profiles.

## 6 Future perspectives

**Figure 3:**
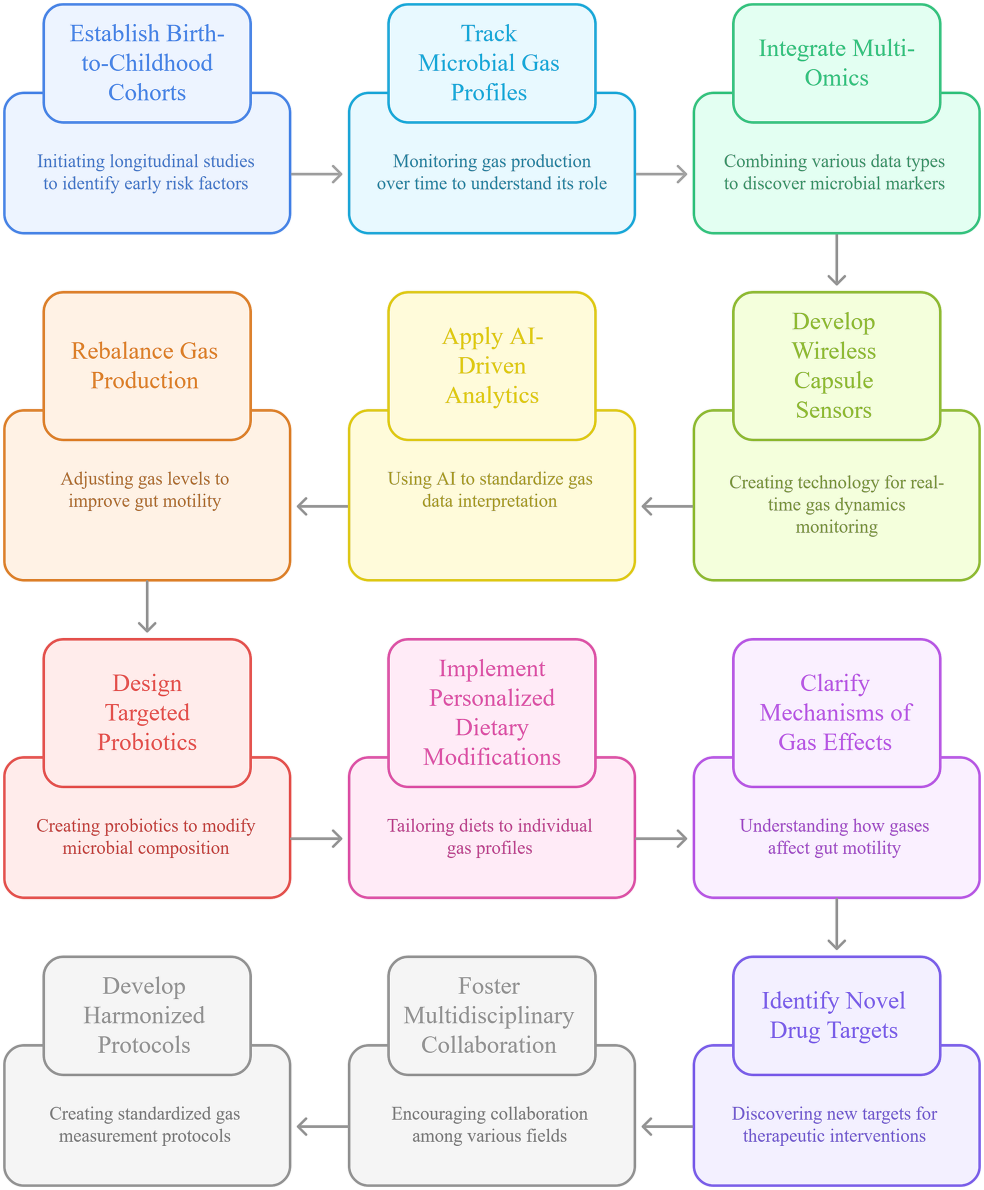
Future research directions and interventional strategies for pediatric functional constipation. This schematic outlines key translational approaches spanning longitudinal cohort studies, advanced gas monitoring technologies, microbiome-targeted therapies, mechanistic investigations, and multidisciplinary collaboration—all aimed at enabling precision management of childhood constipation through modulation of intestinal gas metabolism.

Research into intestinal gas metabolism is poised to fundamentally reshape our understanding and management of PFC. To bridge the gap between mechanistic insights and clinical application, a concerted, multi-pronged strategy is essential. Figure 3 outlines a comprehensive translational roadmap, integrating longitudinal research, technological innovation, targeted therapeutics, and multidisciplinary collaboration to advance gas-targeted precision medicine for children.

Longitudinal, birth-to-childhood cohorts are essential to identify early-life risk factors for PFC. Tracking microbial gas profiles over time can clarify whether CH₄-positive infants or those with altered H_2_ metabolism are predisposed to chronic constipation, supporting early risk stratification. Defining how diet, antibiotics, and psychosocial stress shape gas-producing communities across development may reveal targets for prevention. Integrating multi-omics with quantitative gas measurements can uncover microbial markers that predict PFC onset or therapeutic response, enabling precision prevention and treatment selection.

Advances in non-invasive gas monitoring will improve diagnostic accuracy and access in children. Miniaturized wireless capsule sensors and refined E-nose systems that capture real-time gas dynamics alongside motility parameters, coupled with AI-driven analytics, can standardize interpretation, reduce reliance on specialized laboratories, and broaden availability of precision diagnostics.

Gas-targeted strategies offer a pathway to personalized therapy. Given the dual effects of intestinal gases—CH_4_ slowing transit and H_2_ promoting motility—interventions that rebalance gas production are promising. Probiotics designed to reduce *methanogenic archaea* or enrich selected H_2_-producing taxa, and personalized dietary modifications (for example, low-FODMAP approaches or tailored fiber supplementation) can be aligned to individual gas profiles to limit adverse fermentation. Pharmacologic options, including CH_4_-reducing agents or H_2_-modulating drugs, should be optimized for pediatric use, with dosing guided by serial breath testing. Mechanistic studies linking gas metabolism to motility and the brain–gut axis will reveal new targets. Clarifying how CH_4_ diminishes smooth muscle contractility and how H_2_ augments peristalsis could nominate ion channels or neurotransmitter pathways for drug development. Investigating gasotransmitters such as H_2_S—implicated in motility via TRPV1 and KATP channels—may yield selective inhibitors or donors as novel therapies. Elucidating interactions between gas metabolism and neuroenteric signaling, including serotonin and broader neuroendocrine pathways, can explain stress-related exacerbations and inform combined microbiota-directed and behavioral interventions.

Multidisciplinary integration and standardization are pivotal for clinical translation. Collaboration among gastroenterology, nutrition, microbiology, data science, and engineering can deliver harmonized gas-measurement protocols and pediatric reference ranges. Patient-centered trials testing rational combinations—such as probiotics plus dietary modulation or neuromodulation alongside gas-reducing agents—are needed to address PFC’s multifactorial nature.

In conclusion, uniting longitudinal research, technological advancement, and precision medicine will clarify the interplay among microbial gas production, host physiology, and clinical phenotypes. Such work can shift PFC from a condition that is inconsistently managed to one treated with targeted, personalized strategies, ultimately improving children’s quality of life.
